# Effect of anti-malarial interventions on trends of malaria cases, hospital admissions and deaths, 2005–2015, Ghana

**DOI:** 10.1186/s12936-017-1828-6

**Published:** 2017-04-26

**Authors:** Maru Aregawi, Keziah L. Malm, Mohammed Wahjib, Osae Kofi, Naa-Korkor Allotey, Peprah Nana Yaw, Wilmot Abba-Baffoe, Sylvester Segbaya, Felicia Owusu-Antwi, Abderahmane T. Kharchi, Ryan O. Williams, Mark Saalfeld, Nibretie Workneh, Estifanos Biru Shargie, Abdisalan M. Noor, Constance Bart-Plange

**Affiliations:** 1 World Health Organization, Geneva, Switzerland; 2 World Health Organization, WHO Country Office, Accra, Ghana; 3 World Health Organization, Intercountry Support Team, West Africa, Ouagadougou, Burkina Faso; 4 National Malaria Control Programme, Ministry of Health, Accra, Ghana; 5 AngloGold Ashanti Malaria Control Programme, Obuasi, Ashanti Ghana; 6 The Global Fund to Fight AIDS, Tuberculosis and Malaria, Geneva, Switzerland

**Keywords:** Malaria, Impact, LLIN mass campaign, Indoor residual spraying, Ghana

## Abstract

**Background:**

Since 2005, the Government of Ghana and its partners, in concerted efforts to control malaria, scaled up the use of artemisinin-based combination therapy (ACT) and insecticide-treated nets (ITNs). Beginning in 2011, a mass campaign of long-lasting insecticidal nets (LLINs) was implemented, targeting all the population. The impact of these interventions on malaria cases, admissions and deaths was assessed using data from district hospitals.

**Methods:**

Records of malaria cases and deaths and availability of ACT in 88 hospitals, as well as at district level, ITN distribution, and indoor residual spraying were reviewed. Annual proportion of the population potentially protected by ITNs was estimated with the assumption that each LLIN covered 1.8 persons for 3 years. Changes in trends of cases and deaths in 2015 were estimated by segmented log-linear regression, comparing trends in post-scale-up (2011–2015) with that of pre-scale-up (2005–2010) period. Trends of mortality in children under 5 years old from population-based household surveys were also compared with the trends observed in hospitals for the same time period.

**Results:**

Among all ages, the number of outpatient malaria cases (confirmed and presumed) declined by 57% (95% confidence interval [CI], 47–66%) by first half of 2015 (during the post-scale-up) compared to the pre-scale-up (2005–2010) period. The number of microscopically confirmed cases decreased by 53% (28–69%) while microscopic testing was stable. Test positivity rate (TPR) decreased by 41% (19–57%). The change in malaria admissions was insignificant while malaria deaths fell significantly by 65% (52–75%). In children under 5 years old, total malaria outpatient cases, admissions and deaths decreased by 50% (32–63%), 46% (19–75%) and 70% (49–82%), respectively. The proportion of outpatient malaria cases, admissions and deaths of all-cause conditions in both all ages and children under five also fell significantly by >30%. Similar decreases in the main malaria indicators were observed in the three epidemiological strata (coastal, forest, savannah). All-cause admissions increased significantly in patients covered by the National Health Insurance Scheme (NHIS) compared to the non-insured. The non-malaria cases and non-malaria deaths increased or remained unchanged during the same period. All-cause mortality for children under 5 years old in household surveys, similar to those observed in the hospitals, declined by 43% between 2008 and 2014.

**Conclusions:**

The data provide compelling evidence of impact following LLIN mass campaigns targeting all ages since 2011, while maintaining other anti-malarial interventions. Malaria cases and deaths decreased by over 50 and 65%, respectively. The declines were stronger in children under five. Test positivity rate in all ages decreased by >40%. The decrease in malaria deaths was against a backdrop of increased admissions owing to free access to hospitalization through the NHIS. The study demonstrated that retrospective health facility-based data minimize reporting biases to assess effect of interventions. Malaria control in Ghana is dependent on sustained coverage of effective interventions and strengthened surveillance is vital to monitor progress of these investments.

## Background

Ghana has an area of 238,537 sq km and a population of 27 million [1]. Malaria is intensive and a major public health problem in the majority of the country despite recent progress. Malaria control is integrated into the overall health system through health posts, health centres, district hospitals, regional and referral hospitals complemented by community health planning services (CHIPS). The country is divided into ten administrative regions with three distinct malaria epidemiological strata: (i) savannah malaria characterized by intense and seasonal transmission; (ii) forest malaria characterized by moderate and perennial transmission; and, (iii) coastal malaria with lower but perennial transmission.

Since 2000, the Government of Ghana accelerated malaria control efforts guided by three consecutive national malaria strategic plans covering: first, for the period 2000–2010; second, for 2008–2015; and, an updated one for 2014–2020. Funding for malaria control increased from <US$10 million in 2005 to >US$80 million in 2015 with the support of the Global Fund to Fight AIDS, Tuberculosis and Malaria (GFATM), the President Malaria Initiative (PMI), Department for International Development (DFID), and other development partners. The National Malaria Control Programme (NCMP), scaled up distribution of insecticide-treated nets (ITNs) and artemisinin-based combination therapy (ACT) as key interventions, and IRS in selected districts. For monitoring progress, Ghana’s disease surveillance relies on the District Health Management Information system (DHIMS-2). Data are aggregated by health facility and age with online access to authorized users. The DHIMS-2 is integrated for all diseases with an average reporting completeness of 95% by 2015 from the public and faith-based health centres and hospitals and only 62% from the private sector [2]. Despite this, the data in the DHIMS-2 are currently inadequate to allow reliable analysis of disease trend owing to several limitations, notably on quality of data and reporting completeness caused by factors such as power interruption at facility level, inadequate supervision and compliance by health staff in completing records.

In view of significant investments, the NMCP of Ghana and World Health Organization (WHO) assessed the impact of the interventions on trends of malaria cases and deaths using retrospective data collected from sample hospitals, representing the three epidemiological zones covering the period 2005–2015.

## Methods

### Country context

Routine distribution of ITNs targeting children under 5 years old and pregnant women started in 2003 in 20 pilot districts and was scaled up nationwide during 2004–2010. During 2006–2009, >4.5 million ITNs were distributed through antenatal care (ANC), Child Health weeks, voucher schemes, and commercial sources. However, the strategy was replaced with a long-lasting insecticidal net (LLIN) mass campaign, starting at the end of 2010 with the goal of achieving universal coverage in all ages at a ratio of one net per two persons. During 2010–2012, >12 million LLINs were delivered, and another 12.3 million LLINs during 2013–2015. In 2012, the AngloGold Ashanti Malaria Control Programme (AGAMal) implemented indoor residual spraying (IRS) in seven districts, expanding to 22 districts in five regions (Upper West, Upper East, Ashanti, Western, Central) in 2015. PMI supported five districts for IRS in the Northern region. In total, in 2015, 4.7% of the entire population in the country benefited from IRS. This population also received LLINs.

Parasitological testing of patients of age 5 years and above was primarily limited to hospitals. Since 2010, confirmation of all ages with either microscopy or rapid diagnostic tests (RDTs) has been introduced. Health centres and health posts use RDTs as primary diagnostic tool while higher facilities use microscopy, reserving RDTs for emergency cases, and for situations where microscopy is not functional or caseloads exceed microscopy diagnostic capacity. Therefore, only records of microscopic testing from the hospitals were used for this study.

The country changed its first-line anti-malarial treatment from chloroquine to artesunate–amodiaquine (AS–AQ) in 2004 and added artemether–lumefantrine (AL) and dihydro-artemisinin–piperaquine (DHAP) as alternatives in 2009. Quinine was the principal medicine for severe malaria until 2010, when injectable artesunate was adopted as the drug of choice. The Affordable Medicines facility for Malaria (AMFm), launched in 2011, increased access to ACT in the private sector from 31% in 2010 to 83% in 2011 [3].

For community-based intervention, between 2005 and 2012, nearly 2218 CHPS have been established in the country. The health staff in the CHIPS diagnose with RDTs and treat malaria and assist delivery at the community. However, presumptive treatment of malaria may occur for logistic reasons, when RDTs stock-out.

Prior to 2007, patients of all ages were charged for malaria diagnosis and treatment, which was a major barrier to accessing basic health services for majority of the population. To address the problem of access, the NHIS was initiated in 2003 and became operational in 2005 to subsidize maternal and child health services, which became free in 2007. In the NHIS, urban and rural populations pay a premium of about US$17 and US$11 per year, respectively, and when the cost is beyond what NHIS cover, patients cover the difference. For people whose inability to pay is certified by local authorities, the Government covers all. Despite these provisions, the coverage of the NHIS remains at 38% owing to financial challenges to sustain the scheme, identification of the poor and vulnerable, identification (ID) card management, quality of care, and slow information system [4].

### Data for intervention coverage

Long-lasting insecticidal nets and IRS data were obtained from district records. The proportion of the population potentially protected by LLINs was calculated for each year, assuming each LLIN covered 1.8 persons and lasted 3 years. Prospective district population was derived from the 2010 Ghanaian census, using the United Nations growth rate for Ghana [5, 6]. To measure ACT availability, number of months-ACT stock-out was obtained from records of hospital medical stores. Stock-out was defined when a hospital had no ACT in stock for more than 7 days in a month.

### Data for malaria morbidity and mortality

A WHO standardized protocol (unpublished) was used for hospital data collection. A total of 88 hospitals in three epidemiological zones were randomly sampled (28 in savannah, 30 in forest, 30 in coastal). Although the design was to select 30 representative hospitals from each zone, the savannah had only 28 hospitals and all were sampled. Data abstraction teams consisting of two persons visited each hospital for 2 days.

Monthly summary reports were used as the main source of records for: (i) outpatient all-cause consultations and malaria cases; (ii) inpatient all-cause and malaria admissions, all-cause deaths, malaria deaths, anaemia admissions, and anaemia deaths; and, (iii) laboratory records for microscopically tested and positive cases. Where monthly summary records were missing, register books were used as primary source. Data were collected for two age groups: all ages and under 5 years of age.

Analysis of microscopic tests and confirmed cases was possible only for all ages as records of microscopic results could not be disaggregated by age, outpatient or inpatients. Microscopically confirmed malaria cases, malaria admissions and malaria deaths were the main interest of the analysis. A confirmed malaria case for this study was defined as a blood slide positive for malaria. Malaria admission was defined as a microscopically confirmed inpatient case, diagnosed at discharge as severe malaria. However, some malaria admissions were admitted based on clinical signs suggestive of severe malaria. A malaria death was defined as death attributed to malaria among the admitted cases. All anaemia admissions were assumed to be severe anaemia (<7 mg/dl). The test positivity rate (TPR) was computed by dividing number of positive slides by the total number of slides examined.

To control for other factors that might influence the observed trends, the following additional indicators were computed: (i) non-malaria outpatient consultations (all-cause outpatient cases minus outpatient malaria cases); (ii) non-malaria admissions (all-cause admissions minus malaria admissions); and, (iii) non-malaria deaths (all-cause deaths minus malaria deaths). In addition, data on admitted patients with or without health insurance were extracted from the DHMIS-2 to assess the difference in disease trends among those insured and non-insured. These allowed the assessment of whether changes in malaria cases and deaths were truly attributable to interventions or to changes in healthcare-seeking behaviours or other factors that affect all diseases in a similar way. In addition, the trends of child mortality from population-based surveys for Ghana were also reviewed and triangulated with the trends observed in the hospitals.

To control for the potential effect of climatic changes in trends of malaria during pre- or post-scale-up periods, data on monthly rainfall in mm, minimum and maximum temperature in  °C by region, were obtained from the Ghanaian Meteorology Department.

### Statistical analysis of time trends

For filling missing monthly data, either of the following approaches were applied: (i) if the missing data were in the 1st year, then average the consecutive 2 years of the same month, (ii) if the missing data were in the last year, then average the two preceding years of the same month; and, (iii) if the missing data were in between 2 years, running averages of the preceding and consecutive year for the same month. Because the data points end in June 2015, monthly data were aggregated biannually (two periods for each year) making a total of 21 periods covering January 2005–June 2015.

Assuming all other interventions constant, the effect of an LLIN mass campaign since 2011 was evaluated by comparing the disease trends of the pre-scale up years (2005–2010), i.e., period 1–11 with that of period 12–21 (post-scale up years 2011–2015). Changes on trends were estimated as relative per cent change by comparing the observed value and predicted value at period 21 assuming a continuation of the pre-scale up time trend throughout period 21 if there were no interventions (counterfactual trend). The interpretation of the predicted relative change in percentage at period 21 therefore takes into account the trends (slop) of all the values during 2011 and 2015. This was done using a segmented regression model of an interrupted time series with breakpoint of period 11 [7], an approach that has increasingly become useful to evaluate impact of public health interventions [8, 9] including malaria [10, 11]. The 95% confidence intervals (CI) around effect estimates were computed using the CI around the regression coefficient. A percentage difference with a CI that does not include zero in the range was considered a significant change (*P* < *0.05*). This model corrects for autocorrelation [12, 13] and adjusts the change estimate allowing for: (1) possible time trend of the indicator during the pre-scale-up period, (2) a possible immediate drop or rise of the indicator following the start of the campaign; and, (3) an effect of the campaign on the post-scale up at a given period. In addition, a stratified analysis using segmented regression was applied to compare changes in disease trends in IRS districts (Northern region) and non-IRS districts.

## Results

Eighty-eight district hospitals had complete on outpatient and inpatient data and 70 on labortory data; and all were included in the analysis, each representing a district. Nearly 21,908 missing monthly values, representing 10% of the total 210,709 records for the key indicators, were imputed using either of the approaches described in the methods.

### Interventions

**Fig. 1:**
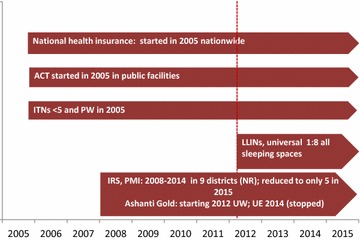
Timing of implementation of anti-malarial interventions in Ghana, 2005–2015 ACT in public sector scaled-up since 2005; ITNs targeting children under five and pregnant women until 2010 and universal coverage of LLINs targeting all population since 2011; IRS in limited number of northern districts since 2007; health insurance scheme since 2005

**Fig. 2:**
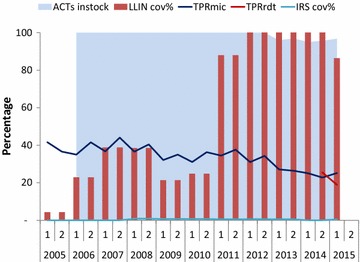
Trends in TPR, percentage of population protected with LLINs, IRS and percentage of health facilities with stock of ACT available by year, 2005–2015, Ghana

Mapping of the interventions is given in Fig. 1. The proportion of population of all ages potentially protected by LLINs increased from nearly 25 in 2005 to 86% in 2015 (Fig. 2). The type of LLIN used in mass and routine distribution during 2005–2015 was a mixture of Permanet^®^ Olyset^®^, Interceptor, and Netprotect. The percentage of the hospitals with available stock of all forms of ACT was 100% during 2006–2012, and >95% during 2013-2015 (Fig. 2). Availability of quinine in stock was 100% during 2005–2012 while it was between 80 and 90% during 2013–2015. After its introduction in 2010, availability of artesunate injectable increased to 83% in the first half of 2015.

### Trends on the malaria outpatient cases, inpatient and deaths

**Fig. 3:**
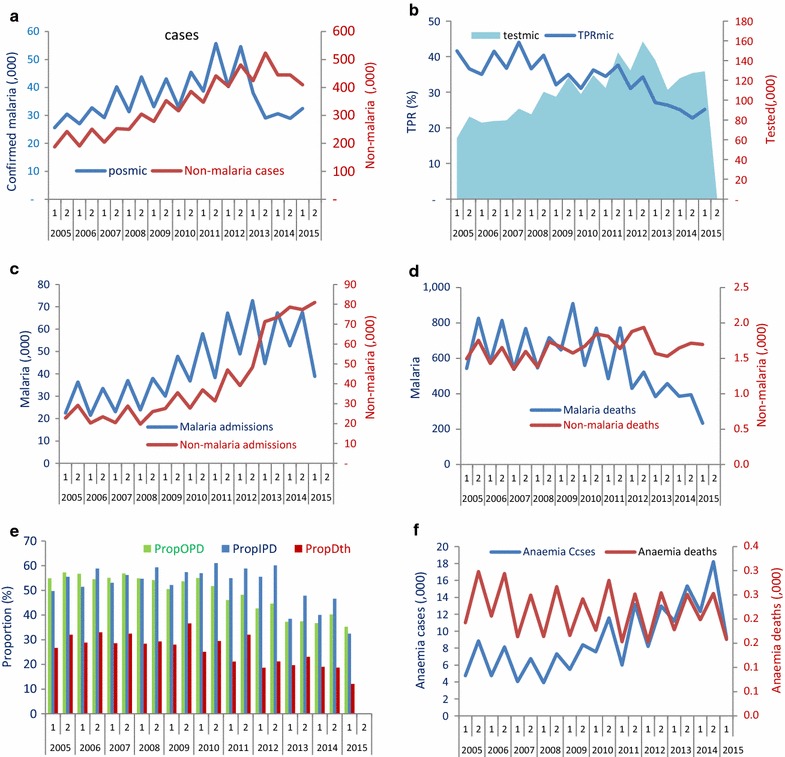
Biannual trends of cases and deaths in 88 hospitals, 2005–2012, Ghana. **a** Trends of confirmed malaria cases versus non-malaria outpatient cases; **b** TPR versus cases tested with microscopy; **c** malaria admissions versus non-malaria admissions; **d** malaria deaths *versus* non-malaria deaths; **e** proportion of outpatient malaria cases (PropOPD), admissions (PropIPD), and deaths of all-cause conditions (PropDth); **f** severe anaemia admissions and anaemia deaths

Among all ages, the number of suspected malaria cases declined from a 6-months average count of 635,161 during 2005–2010 (period 1–10) to 581,906 in the first half of 2015 (period 21), with an observed decrease of only 8%, but significant predicted decrease of 57% (95% CI, 47–66%) during 2011–2015 compared to the trend of 2005**–**2015 (assuming continuation of the trend at the pre-scale-up level). The number of microscopically confirmed cases had little change in observed values but a significant predicted decrease of 53% (28–69%), while the number of microscopic tests had an observed increase and little change in predicted values. The TPR in all ages declined by 30% (observed) and a predicted decrease of 41% (52–75%) nationwide during the same period. Malaria admissions showed 52% increase in observed trends and insignificant predicted change, 39% (0–63%). Malaria deaths fell by 49% in observed and predicted trends significantly by 65% (52–75%) (Table 1; Fig. 3a–d). The number of anaemia inpatient cases showed an observed increase of 126% but insignificant predicted trend. Anaemia-related deaths increased by 47% with insignificant predicted changes. The proportion of outpatient malaria cases, malaria admissions and malaria deaths of all-cause conditions in the hospitals showed an observed decrease of 36,17 and 52% respectively and a significant predicted decline of 32% (21–42%), 41% (23–54%) and 61% (48–70%), respectively (Table 1; Fig. 3e).

**Table 1:** Nationwide percentage change of malaria and non-malaria related indicators in post-scale-up years compared to pre-scale-up period, by age, 88 hospitals, in a given number of health facilities with complete data 2005–2015, Ghana

No of HFs	Indicator	All ages				Under 5			
		Baseline	Observed values	Observed relative change (%)	Predicted relative change compared to baseline (%)	Baseline	Observed values	Observed relative change (%)	Predicted relative change compared to baseline (%)
		Ave (2005−2010) P1	2015 P1	2015 P1	2015 P1	Ave (2005−2010) P1	2015 P1	2015 P1	2015 1
88	Outpatient malaria cases	635,161	581,906	−8	−57 (−66 to −47)^†^	145,419	155,677	7	−50 (−63 to −32)^†^
	Malaria admissions	60,070	91,425	52	−39 (−63 to 0)^†^	26,274	38,857	48	−46 (−75 to 19)
	Malaria deaths	1229	632	−49	−65 (−75 to −52)^†^	569	234	−59	−70 (−82 to −49)^†^
	Anaemia inpatients	10,611	24,004	126	1 (−68 to 221)	5082	9245	82	−17 (−75 to 179)
	Anaemia deaths	456	672	47	38 (−19 to 135)	178	158	−11	−24 (−63 to 59)
70	Tested (Mic)	247,750	374,394	51	−22 (−54 to 34)	85,514	129,092	51	
	Positive (Mic)	87,610	92,383	5	−53 (−69 to −28)^†^	29,859	32,476	9	
	Test positivity rate (mic)	35	25	−30	−41 (−57 to −19)^†^	36	25	−29	
88	Proportion of outpatients	36	23	−36	−32 (−42 to −21)^†^	54	35	−35	−30 (−37 to −21)^†^
	Proportion of inpatients	25	21	−17	−41 (−54 to −23)^†^	53	32	−39	−49 (−57 to −40)^†^
	Proportion of deaths	15	7	−52	−61 (−70 to −48)^†^	28	12	−56	−59 (−72 to −40)^†^
	Non-malaria outpatient	1706,037	2439,358	43	−8 (−28 to 17)	237,858	409,470	72	−28 (−48 to 1)
	Non-malaria inpatients	174,948	344,964	97	24 (−11 to 72)	23,071	80,890	251	94 (−7 to 303)
	Non-malaria deaths	7175	8340	16	−1 (−23 to 28)	1497	1697	13	−8 (−36 to 32)

CI with symbol “†” is statistically significant

In children under 5 years old, total malaria outpatient cases (both confirmed and presumed cases) had little change in observed values but a significant predicted decrease of 50% (32–63%). Malaria deaths had an observed 59% decrease and a significant predicted decline of 70% (49–82%). The number malaria admissions had an observed increase of 48% and insignificant predicted change of 46% (19–75%); the proportion of outpatient malaria cases, admissions and deaths of all-cause conditions showed an observed decline of 35, 39 and 56% respectively; and a significant predicted decline of 30% (21–37), 49% (40–57%) and 59% (40–72%) (Table 1). The number of anaemia inpatient cases showed an observed increase of 82% but insignificant predicted trend. Anaemia-related deaths decreased by 11% with insignificant predicted changes.

Concomitantly, the non-malaria outpatient cases, non-malaria inpatient and non-malaria deaths in both all ages and children under five generally increased or remained unchanged during the same period (Fig. 3; Table 1).

### Population based mortality

**Fig. 4:**
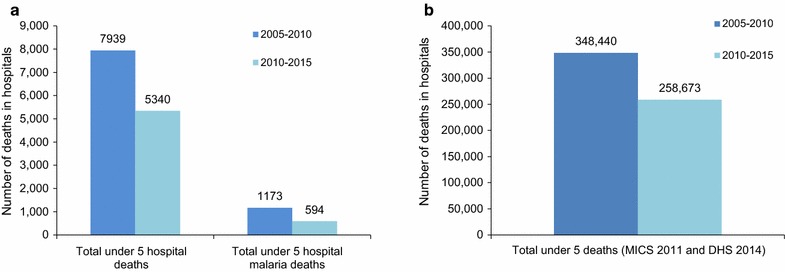
All-cause deaths and malaria deaths in children under five at hospital and population level during 2005–2015. **a** Under five all-cause and malaria deaths in hospitals; **b** Under five all-cause deaths at population level

All-cause mortality for children under 5 years of age in household surveys declined from 105 per 1000 live births during 2004–2008 [16], to 82 per 1000 live births during 2009–2011 [15], and to 60 per 1000 during 2010–2015 [16] representing at least a decline of 43% between 2008 and 2014. Five-year cumulative all-cause deaths in under 5 years in the 88 hospitals declined from 7939 during 2005–2010 to 5340 during 2011–2015 (33% decline). During the same period, hospital malaria deaths declined by 49% (1173 to 594). Total all-cause child deaths at population level decreased from 348,440 to 258,673 during the same period (26% decline). Deaths in the 88 hospitals represented only 1% of all the deaths at population level attributed from the household surveys (Fig. 4).

### Trends among the insured and non-insured by NHIS

**Fig. 5:**
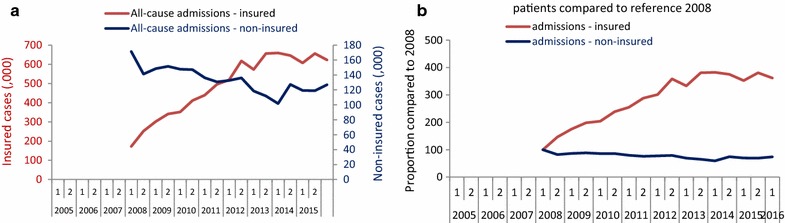
Trends of all-cause admissions among insured and non-insured patients (Ghana MoH). **a** All-cause admissions in absolute values; **b** all-cause admissions compared (indexed) to baseline 2008

Nationwide hospital data from the DHIMS-2 were used to discern the trends of all-cause admissions among the insured and non-insured. All-cause admissions among the insured in all ages increased by 99% while it decreased by 21% among the non-insured patients (Fig. 5).

### Spatial differences

The confirmed malaria cases in the three epidemiological zones (coastal, forest, savannah) decreased significantly by 16% (16–40%), 66% (30–83%), and 55% (9–77%), respectively. TPR decreased significantly by 33% (7–58%) in coastal and by 37% (2–61%) in savannah while the change in forest was insignificant. Malaria admissions in all ages showed little or insignificant change in forest and savannah but decreased significantly by 40% in coastal (14–58%). Malaria deaths declined significantly by >64% in all the three zones. For children under five, malaria admissions either remained unchanged or increased while malaria deaths fell by >57% across all three strata. Observed trends of inpatient anaemia cases in Coastal, Forest and Savannah increased by 32, 216 and 136% respectively while the anaemia-related deaths increased by only 37, 16 and 106%, respectively. All predicted trends of anaemia cases and deaths were statistically insignificant (Table 2).

**Table 2:** Percentage change in malaria cases, admissions and deaths, and test positivity rate in 2015 period 1 compared to pre-scale-up period (2005–2010) period 1, by epidemiological zone in given number of health facilities (HF) with complete data 2005–2015, Ghana

Stratum	No of HFs	Indicator	All ages				Under 5			
			Baseline	Observed values	Observed relative change (%)	Predicted relative change compared to baseline (%)	Baseline	Observed values	Observed relative change (%)	Predicted relative change compared to baseline (%)
			Ave (2005−2010) P1	2015 P1	2015 P1	2015 P1	Ave (2005−2010) P1	2015 P1	2015 P1	2015 1
Coastal	30	Outpatient malaria cases	215,920	193,102	−11	−57 (−71 to −37)^†^	42,069	43,377	3	−52 (−70 to −25)^†^
		Malaria admissions	17,722	17,607	−1	−40 (−58 to −14)^†^	7596	8284	9	−26 (−49 to 7)
		Malaria deaths	505	181	−64	−68 (−84 to −35)^†^	192	56	−71	−57 (−76 to −22)^†^
		Anaemia inpatients	3773	4985	32	14 (−37 to 107)	1509	1879	25	4 (−55 to 144)
		Anaemia deaths	205	281	37	74 (12 to 171)	82	64	−21	39 (−8 to 109)
		Tested (Mic)	89,297	136,568	53	25 (−31 to 129)	46,182	135,576	194	
		Positive (Mic)	29,615	42,550	44	−16 (−40 to 16)	24,319	57,544	137	
		Test positivity rate (mic)	34	31	−9	−33 (−58 to 7)	54	42	−22	
		Proportion of outpatients	29	21	−28	−23 (−43 to 2)	46	30	−34	−31 (−43 to −17)^†^
		Proportion of inpatients	21	16	−24	−34 (−55 to −3)^†^	47	34	−28	−36 (−46 to −24)^†^
		Proportion of deaths	17	5	−72	−76 (−90 to −42)^†^	29	7	−77	−73 (−86 to −47)^†^
		Non-malaria outpatient	718,045	871,851	21	−45 (−64 to −17)^†^	68,351	86,222	26	−42 (−68 to 3)
		Non-malaria inpatients	67,327	94,489	40	0 (−17 to 21)	8499	16,124	90	61 (14 to 128)
		Non-malaria deaths	2493	3622	45	56 (8 to 127)	462	769	66	100 (22 to 229)
Forest	30	Outpatient malaria cases	235,523	197,145	−16	−57 (−65 to −48)^†^	50,020	51,668	3	−46 (−62 to −25)^†^
		Malaria admissions	19,887	31,406	58	−27 (−46 to −1)^†^	8162	12,748	56	−44 (−70 to 5)
		Malaria deaths	222	89	−60	−67 (−78 to −49)^†^	101	31	−69	−81 (−88 to −69)^†^
		Anaemia inpatients	3597	11,377	216	32 (−9 to 92)	1887	4280	127	8 (−36 to 81)
		Anaemia deaths	142	165	16	−20 (−63 to 72)	43	42	−2	−45 (−77 to 31)
	24	Tested (Mic)	28,702	29,626	3	−70 (−89 to −15)^†^	10,518	25,094	139	
		Positive (Mic)	6184	4896	−21	−66 (−83 to −30)^†^	3152	3448	9	
		Test positivity rate (mic)	22	17	−26	13 (−32 to 87)	30	14	−54	
	30	Proportion of outpatients	37	19	−49	−50 (−60 to −38)^†^	52	29	−44	−46 (−57 to −31)^†^
		Proportion of inpatients	26	25	−2	−28 (−49 to 1)	51	41	−20	−44 (−53 to −31)^†^
		Proportion of deaths	7	4	−46	−41 (−61 to −12)^†^	14	8	−40	−40 (−70 to 20)
		Non-malaria outpatient	634,600	1037,746	64	−14 (−33 to 11)	93,083	173,324	86	−3 (−22 to 21)
		Non-malaria inpatients	56,415	91,878	63	15 (−26 to 79)	7704	18,677	142	76 (16 to 166)
		Non-malaria deaths	2795	2132	−24	−41 (−64 to −5)^†^	613	335	−45	−66 (−80 to −42)^†^
Savannah	28	Outpatient malaria cases	183,718	191,659	4	−59 (−73 to −39)^†^	53,330	60,632	14	−51 (−70 to −20)^†^
		Malaria admissions	22,460	42,412	89	−48 (−77 to 19)	10,517	17,825	69	−54 (−85 to 41)
		Malaria deaths	502	362	−28	−64 (−79 to −38)^†^	276	147	−47	−70 (−86 to −39)^†^
		Anaemia inpatients	3241	7642	136	−30 (−94 to 659)	1686	3086	83	−45 (−94 to 370)
		Anaemia deaths	110	226	106	59 (−41 to 329)	54	52	−4	−49 (−85 to 79)
	18	Tested (Mic)	21,471	40,977	91	−28 (−55 to 16)	24,823	33,395	35	
		Positive (Mic)	8910	13,435	51	−55 (−77 to −9)^†^	11,613	10,697	−8	
		Test positivity rate (mic)	42	33	−21	−37 (−61 to 2)	47	32	−32	
	28	Proportion of outpatients	46	33	−28	−15 (−34 to 9)	69	50	−27	−5 (−36 to 42)
		Proportion of inpatients	30	21	−30	−51 (−66 to −30)^†^	61	28	−54	−55 (−71 to −29)^†^
		Proportion of deaths	21	12	−41	−57 (−71 to −35)^†^	40	20	−50	−59 (−71 to −41)^†^
		Non-malaria outpatient	396,294	561,263	42	−52 (−67 to −28)^†^	67,199	110,711	65	−52 (−76 to 0)^†^
		Non-malaria inpatients	51,207	158,597	210	44 (−19 to 156)	6869	46,089	571	89 (−70 to 1095)
		Non-malaria deaths	1887	2586	37	2 (−26 to 40)	421	593	41	14 (−28 to 82)

Negative percentages indicate decrease in the indicator while positive percentages indicate increase during the post-scale up period (2011–2015) compared to the pre-scale-up period

CI with symbol “†” is statistically significant

### Difference in IRS and non-IRS districts

In the regions where IRS was implemented during 2006–2015 (24 districts of Ashanti, Northern, Upper East, Upper West, and West Region), the TPR in all ages decreased significantly by 89% (77–95%); malaria admissions and deaths decreased significantly by 68% (21–87%) and 88% (71–95%), respectively. On the other hand, the decrease in trends of malaria indicators in the non-IRS districts (34 within the same regions) was smaller. The TPR decreased only by 38% (16–54%); malaria admissions showed little change, 35% (−15 to 63%), and malaria deaths decreased by 44% (16–62%). The decreases in proportion of malaria outpatients, inpatients and deaths of all-cause conditions were much higher in the IRS districts compared to the non-IRS districts (Table 3).

**Table 3:** Percentage change in malaria admissions and deaths, and test positivity rate in 2015 compared to pre-scale-up period (2005-2010), in IRS-districts (24), and non-IRS districts (36) in Ashanti, Northern, Upper East, Upper West, and Western Regions, Ghana, 2005–2015

Stratum	Indicator	IRS, all ages				Non−IRS, all ages			
		Baseline	Observed values	Observed relative change (%)	Predicted relative change compared to baseline (%)	Observed			Predicted relative change compared to baseline (%)
		Ave (2005−2010) P1	2015 P1	2015 P1	2015 p1	Ave (2005−2010) P1	2015 P1	Change % 2015 P1	2015 1
National	Outpatient malaria cases	167,030	143,774	−14	−47 (−79 to 30)	858,019	800,050	−7	−58 (−69 to−44)^†^
	Malaria admissions	16,103	17,812	11	−68 (−87 to −21)^†^	94,207	172,697	83	−35 (−63 to 15)
	Malaria deaths	424	166	−61	−88 (−95 to −71)^†^	1608	1172	−27	−44 (−62 to −16)^†^
	Aneamia inpatients	1797	6954	287	17 (−74 to 422)	13,090	38,379	193	9 (−85 to 669)
	Aneamia deaths	107	152	42	−38 (−80 to 90)	542	829	53	52 (−21 to 194)
	Tested (Mic)	76,758	143,162	87	110 (−69 to 1313)	302,938	457,757	51	−48 (−82 to 48)
	Positive (Mic)	19,670	17,552	−11	−76 (−94 to 2)	125,591	130,379	4	−68 (−88 to −17)^†^
	Test positivity rate (mic)	27	12	−54	−89 (−95 to −77)^†^	42	28	−32	−38 (−54 to −16)^†^
	Proportion of outpatients	35	26	−26	−11 (−61 to 100)	44	29	−35	−30 (−35 to −23)^†^
	Proportion of inpatients	25	17	−30	−56 (−71 to −33)^†^	32	25	−23	−37 (−54 to −13)^†^
	Proportion of deaths	18	6	−66	−87 (−96 to −63)^†^	21	12	−42	−41 (−56 to −20)^†^
	Non-malaria outpatient	470,715	544,484	16	−38 (−57 to −9)^†^	1822,697	2,638,677	45	−38 (−54 to −15)^†^
	Non-malaria inpatients	48,640	86,050	77	−6 (−51 to 83)	194,843	517,232	165	27 (−22 to 107)
	Non-malaria deaths	1977	2552	29	46 (−28 to 196)	6164	8587	39	4 (−32 to 59)

Negative percentages indicate decrease in the indicator while positive percentages indicate increase during the post-scale up period (2011–2015) compared to the pre-scale-up period

CI with symbol “†” is statistically significant

### Effect of rainfall anomalies and seasonality

**Fig. 6:**
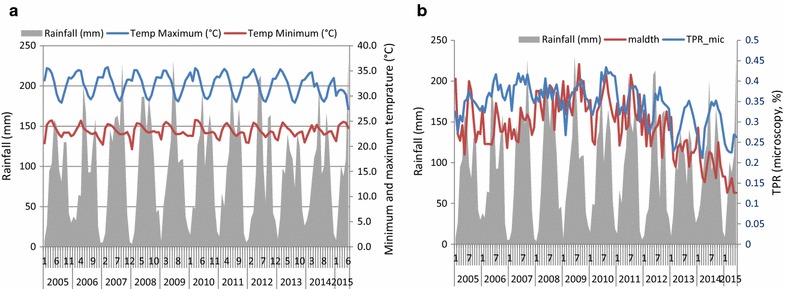
Rainfall, minimum temperature (°C), maximum temperature, trends of malaria deaths and TPR. **a** Rainfall (mm), minimum and maximum temperature (°C); **b** rainfall, malaria deaths and TPR

There were no noticeable anomalies in rainfall and temperature that could plausibly affect the normal seasonal trends of malaria cases and deaths (Fig. 6a). Mean minimum and maximum rainfall was 82 and 118 mm, respectively. The mean minimum temperature during pre- and post-scale-up was similar, 22.0 °C (21.5–22.8 °C) and 22.9 °C (22.2–23.8 °C), respectively; the mean maximum temperature was 31.8 °C (30.3–33.3 °C) and 31.6 °C (27.8–33.8 °C), respectively (Fig. 6b). A time-series regression on the effect of the three climatic factors on malaria deaths and TPR, as an example, showed little effect from variations in rainfall and maximum temperature. Minimum temperature appeared to have significant effect on reducing the trends of both indicators (malaria deaths and TPR) controlling for effect of time (*P* < *0.05*) (Table 4).

**Table 4:** Time-series regression of climatic factors on the trends of malaria deaths and test positivity rate

	Coefficient.	Std. err.	t	P > t	(95% CI)	
					Low	High
Malaria deaths						
Rainfall	0.000181	0.002017	0.09	0.930	−0.00414	0.004507
Min temperature	−0.17246	0.041664	−4.14	0.001	−0.26182	−0.0831
Max temperature	0.003815	0.022157	0.17	0.866	−0.04371	0.051337
Constant	10.90383	0.604018	18.05	0	9.608344	12.19933
Test positivity rate						
Rainfall	0.003097	0.003024	1.02	0.364	−0.0053	0.011492
Min temperature	−0.16048	0.044244	−3.63	0.022	−0.28332	−0.03764
Max temperature	−0.00271	0.019038	−0.14	0.894	−0.05556	0.050149
Constant	7.395645	0.677037	10.92	0	5.515888	9.275402

## Discussion

Between 2002 and 2009, mixed LLIN distribution models, including free distribution to targeted groups, subsidized LLINs through the public, private sector and NGOs, and full-cost sales, resulted in moderate increase of ITN ownership in 2008 [14]. The mass free distribution of LLINs from 2010–2012 resulted in a significant improvement in LLIN ownership and use. The percentage of households that owned at least one ITN increased from 18.7% in 2003 [17], 48.3% in 2011 [15], to 96% in 2012 [18, 19], 68% in 2014 [15]. Correspondingly, the proportion of children under five who slept under an ITN increased from 22% in 2003 to 39% in 2011 [15], and 77% in 2012 [18, 19], while it was lower (47%) in 2014 [16]. Since 2005, ACT was readily available in all public sectors.

Following the LLIN campaigns in 2010, this study in 88 hospital data showed a 53% decline in trends of microscopically confirmed malaria cases against a backdrop of increased or constant testing, 65% decrease in malaria deaths and 41% decrease in TPR in all ages. The number of outpatient malaria cases, and malaria deaths in children under 5 years, decreased by 50 and 70%, respectively. Malaria admissions increased during the study period. In all three epidemiological zones, outpatient malaria cases decreased by 50%, malaria deaths by 60%, and TPR by >30%. Malaria admissions in children under five increased during 2011–2015 across all epidemiological zones. The decrease in the main malaria indicators was higher in the regions where IRS was employed, i.e., malaria admissions (68%), malaria deaths (88%) and TPR (89%). Whereas, in non-IRS regions, malaria admissions increased by 35%, malaria deaths and TPR decreased by only 44 and 38%, respectively. All the key malaria indicators decreased significantly across all age groups and regions with different levels of baseline malaria burden in the face of increased healthcare utilization. The paradox with the increase in malaria admissions (particularly in children under five) during the post-scale-up period (2011–2015) can be explained by the increased service availability or access to free admission owing to the provision of NHIS. The twofold increase (126%) in the number of inpatient anaemia cases shows the strong association with malaria admissions accessing free inpatient services. The increase in inpatient anaemia was particularly prominent in the Forest and Savannah zones (Table 2). Although the coverage of the NHIS has been sub-optimal (38%) [20], the scheme would have increased the number of poor malaria patients with severe malaria hospitalized for free who otherwise would have not been admitted. This may have underestimated the accrual impact on the trend of admissions. This argument is supported by Fig. 5, in which the trend of all-cause admissions among the insured increased while the admissions among non-insured were stable or decreasing during 2008–2015. There was no additional data to further explain the cause of disparities although it is plausible to assert that large segments of the population contributing to the NHIS are likely to be those benefiting from free services, including outpatient, admissions and laboratory services [21]. Service-wise, preferential behaviours seem to have emerged as per reported in one study in which the service providers complained of the increased workload and favoured the uninsured (for instant payers) [22]. Furthermore, the NHIS has different cost estimates for different diseases in distributing its budget and supplies with substantial cost and budgetary implications to the hospitals and districts. From the data available, it was not clear to conclude if the NHIS has affected the behaviour of health staff in hospitals in favourably assigning malaria as a cause of admissions of febrile severe diseases regardless of test results or severity.

Although data on population level parasite prevalence for the pre-LLIN campaign was unavailable, the average TPR during post-scale-up period (25%) observed in the health facilities (Table 2) was comparable with the nationwide malaria parasite prevalence in the multiple indicator survey (MICS) in children under 5 years (27.5%) in 2011 [23] and DHS (26.7%) in 2014 [16]. No significant effect of rainfall or temperature on the trends of malaria was identified. Although unmeasured factors may have contributed to the increase or decrease in most malaria indicators in health facilities in Ghana, available data indicated that the decline in malaria deaths coincided with the latest scale up of LLIN mass campaign. The effect of malaria control activities using facility data has been reported previously in other African countries with high transmission, including Rwanda [24], Zanzibar [25], The Gambia [26], Sao Tome and Principe [27], and Ethiopia [28]. In these countries, similar effect of scaled-up malaria control interventions were documented. Other recent sub-national level studies on the effect of the interventions in areas of high transmission include: Angola [29], Bioko Island in Equatorial Guinea [30], Tanzania [31], and Zambia [32].

All-cause mortality for children under 5 years in household surveys showed a decline from 105 per 1000 live births during 2004–2008 (DHS 2008), to 82 per 1000 live births during 2009–2011 (MICS 2011), and 60 per 1000 live births representing at least a decline of 43% between 2008 and 2014. The decrease in malaria deaths may have contributed to the decline in all-cause deaths in children under five, consistent to previous observations [33]. However, the low specificities of verbal autopsies for disease-specific cause of deaths make it impossible to precisely estimate the attribution of reduction in malaria mortality to the decline in all-cause under-five mortality as per that shown in a previous study for other African countries with stable malaria [34].

### Limitations

Caution has to be made when interpreting the results of such health facility-based data often affected by changes in service utilization and reporting completeness [35]. The analysis in this study addressed this problem through data collection from selected 88 health facilities (representing three epidemiological zones) with complete reporting and parasitological testing covering the study periods. Other limitations include: (i) inability to assess the trends in peripheral facilities such as health centres and CHIPs as testing with RDTs in these facilities started only after 2010; (ii) inability to assess the trends in private health facilities; (iii) incomplete data on RDT testing in the hospitals. Although the use of RDTs in the hospitals is limited only in the absence of microscopist and confirmation of highly suspicious severe cases, such usage may have affected the testing rate or patterns of use of microscopic diagnosis; and, (iv) inability of the data to show disaggregated trends of insured and non-insured malaria admissions.

## Conclusion

Five years (since 2011) after universal distribution of LLINs targeted all ages while maintaining high coverage of all other interventions during both pre- and post-scale up periods, the study showed substantial decrease across all malaria indicators including: confirmed cases, test positivity rate and malaria death. Declines in malaria deaths were higher in children under five than in older age groups. The trends in malaria indicators seen at the health facilities evidently reflected the steep downward trend of all-cause child mortality at the population level. The climatic effect on the time trend of malaria indicators in Ghana seems to be significantly related to fluctuation in minimum temperature, the importance of which is reported elsewhere [36, 37]. Increased access to health services, complemented with the NHIS, have encouraged malaria patients to seek health services in hospitals in recent years more than before, underestimating the true impact of anti-malarial interventions. The study indicates: (i) the need for addressing the inequity that arises from NHIS among the insured and non-insured populations, and case definition of disease and the criteria for budgetary allocations. Maintaining high coverage of LLINs in all ages and IRS (where implemented) is critical to avoid resurgence of malaria, which is a real threat when intensity of anti-malarial interventions is disrupted or scaled down. The Ghana Health Service need to address the critical deficiencies the surveillance system is currently experiencing, including: (i) revision of the DHIM-2 to address disease-specific needs, including disaggregated data on effect of the NHIS on testing and malaria deaths; and, (ii) conduct regular supervision, data review and quality audit to ensure data completeness and timeliness.

## Abbreviations

ACT: artemisinin-based combination therapy
AL: Artemether–lumefantrine
AGAMal: AngloGold Ashanti Malaria Control Programme
ANC: antenatal care
AMFm: Affordable Medicines Facility for Malaria
CHPS: community health planning services
CI: confidence interval
DHAP: dihydro-artemisinin–piperaquine
DHIMS2: District Health Information System version 2
GMoH: Ghana Ministry of Health
HFs: Health facilities
NMCP: National Malaria Control Programme
IRS: indoor-residual spraying
LLIN: long-lasting insecticidal net
NHIS: National health insurance scheme
PropOPD: malaria as a proportion of all-cause outpatient cases
PropIPD: malaria as a proportion of all-cause admissions
PropDth: malaria as a proportion of all-cause deaths
PMI: President’s Malaria Initiative
RDT: rapid diagnostic test
TPR: test positivity rate
WHO: World Health Organization
